# Associations between urinary phthalate concentrations and antral follicle count among women undergoing *in vitro* fertilization

**DOI:** 10.3389/fendo.2023.1286391

**Published:** 2024-01-08

**Authors:** Yangcheng Yao, Yaoyao Du, Na Guo, Fenghua Liu, Taoran Deng, Yufeng Li

**Affiliations:** ^1^ Reproductive Medicine Center, Tongji Hospital, Tongji Medical College, Huazhong University of Science and Technology, Wuhan, China; ^2^ Reproductive Medicine Center, Guangdong Women and Children Hospital, Guangzhou, China

**Keywords:** phthalate, urine, antral follicle count, infertile women, *in vitro* fertilization

## Abstract

**Background:**

Phthalates are ubiquitously used in a variety of products and have an adverse effect on folliculogenesis. However, previous epidemiological studies on the associations between phthalate exposure and antral follicle count (AFC) produced conflicting results. The present study aimed to evaluate the associations between urinary phthalate metabolite concentrations and AFC among women undergoing *in vitro* fertilization (IVF).

**Methods:**

We collected 525 urine samples and measured 8 phthalate metabolites from IVF patients. Poisson regression models were conducted to evaluate the associations between phthalate metabolite concentrations and AFC. In addition, participants were stratified into a younger group (< 35 years) and an older group (≥ 35 years) to explore the potential effect modification by age. We also performed sensitivity analyses by restricting our analyses to only infertile women diagnosed with tubal factor infertility to test the robustness of the results.

**Results:**

Significant positive associations were observed among urinary MBP, MEOHP and ∑PAEs concentrations and AFC after adjusting for age, BMI, year of study and infertility diagnosis. Compared with women in the first tertile, women in the third tertile of MBP and MEOHP had 7.02% (95% CI: 1.18%, 12.9%) and 8.84% (95% CI: 2.83%, 14.9%) higher AFC, respectively, and women in the second and third tertiles of ∑PAEs had 6.19% (95% CI: 0.37%, 12.0%) and 9.09% (95% CI: 3.22%, 15.0%) higher AFC, respectively. In addition, MBP, MEOHP and ∑PAEs also had significant positive associations with AFC in trend tests for dose-response. In the age-stratified analysis, we found a stronger relationship between phthalate metabolite concentrations and AFC among older women and an inverse association among younger women. We observed similar results in the sensitivity analyses.

**Conclusion:**

We found positive associations between phthalate exposure and AFC, which support the idea that phthalate exposure may accelerate primordial follicle recruitment and lead to higher AFC in women undergoing IVF. More studies are needed to better understand their relationships.

## 1 Introduction

Infertility is an ongoing reproductive health problem around the world, and exposure to environmental contaminants is an important factor (1, 2). Phthalate esters (PAEs) are a group of synthetic compounds that are abundantly used as plasticizers or solvents in a variety of products, such as polyvinylchloride, building and finishing materials, personal care products, cosmetics, toys, food packages, medications and medical devices (3). Phthalates in products can be released into the environment due to nonchemical bonds, and human exposure occurs through inhalation, ingestion and dermal absorption (4). Phthalates are rapidly metabolized into monoesters once they enter the human body, in which they have stronger biological activity (5). The metabolites are excreted mainly via urine and have a half-life less than 24 hours (6), therefore, urinary metabolite concentrations are generally used to represent body phthalate exposure levels (7).

Phthalates are endocrine disruptors characterized mainly by their reproductive and developmental toxicity (8). According to the results of several animal studies, they can disrupt ovarian development, inhibit follicle growth, and impair oocyte maturation and embryo development (9–14). Epidemiological studies found that phthalate exposure was associated with adverse reproductive outcomes, such as decreased oocyte retrieval, mature oocytes, fertilized oocytes, good-quality embryos, clinical pregnancy rate and live birth rate (15–18).

Antral follicle count (AFC) is a critical value used to evaluate women’s fecundity, and is defined as the sum of 2-10 mm follicles in both ovaries as observed on ultrasound in the early follicular phase (menstrual days 2-4) (19). As the earliest acquirable follicle parameter in the reproductive clinic, AFC was routinely measured during the infertility treatment period for ovarian reserve assessment, infertility diagnosis, and treatment strategy determination, and AFC was also found to be associated with reproductive outcomes (20, 21).

Phthalates have been shown to reduce antral follicle number in mice (22) and inhibit antral follicle growth in *in vitro* culture (23). However, phthalate exposure in humans is complicated and continuous, and the results among human studies are controversial. Messerlian et al. reported that urinary phthalate metabolite concentrations were adversely associated with AFC (24), and Li et al. found both positive and negative associations between phthalate metabolite concentrations in serum and AFC (25). Additionally, we observed positive dose-response associations between urinary phthalate metabolites and AFC in our previous study (26). In mammals, the follicle cycle starts from primordial follicle activation and undergoes a series of developments until atresia or ovulation; thus, more AFC may suggest more follicle recruitment from the beginning. It has been reported that di(2-ethylhexyl) phthalate (DEHP) can accelerate primordial follicle recruitment in mice, leading to a lower proportion of primordial follicles and a higher proportion of developing follicles in ovaries (27–29). In the present study, we enlarged the sample size and reanalyzed the association between phthalate exposure and AFC among women undergoing *in vitro* fertilization (IVF). We also performed stratified analyses to explore potential effect modification by age and conducted sensitivity analysis to test the strength of our results.

## 2 Materials and methods

### 2.1 Participants

To explore the potential effects of phthalate exposure on reproductive health, women who sought infertility treatment were recruited at two separate times at the Reproductive Medicine Center of Tongji Hospital, Wuhan, China, as described previously: from July to August 2014 (30) and from November to December 2016 (31). Briefly, women aged from 20 to 45 years who were infertile and with indications for IVF or intracytoplasmic sperm injection were eligible. Subjects who had an ovariectomy history, other iatrogenic injuries, or health conditions such as autoimmune diseases, congenital gonadal dysplasia, endocrine diseases and sexually transmitted diseases were excluded. Participants who provided a urine sample for phthalate metabolite detection and had AFC data within a 4-month period before enrollment were included in this study. Information on age, height, weight, smoking status and ethnicity was collected at enrollment. These studies were approved by the Ethics Board of Tongji Hospital, and informed consent was obtained for all participants.

### 2.2 Sample collection and measurement

On the day of ovum pick-up surgery, urine samples were collected and transferred to the laboratory immediately, aliquoted and frozen at −80 °C. The concentrations of mono-ethyl phthalate (MEP), mono-methyl phthalate, mono-n-butyl phthalate (MBP), mono(2-ethylhexyl) phthalate (MEHP), mono-benzyl phthalate, mono(2-ethyl-5-oxohexyl) phthalate (MEOHP), mono(2-ethyl-5-hydroxyhexyl) phthalate (MEHHP) and mono-n-octyl phthalate (MOP) were analyzed by high-performance liquid chromatography and tandem mass spectrometry as described in our previous study (26, 32). The limits of detection ranged from 0.01-0.04 μg/L. Values for metabolites with concentrations less than the LOD were assigned with LOD/ $\sqrt{2}$ . Concentrations of creatinine were measured using clinical chemistry analyzers (17).

### 2.3 Infertility data and AFC measurement

AFC, which was measured on days 2-4 of the menstrual cycle by transvaginal ultrasound, and other clinical data (e.g., infertility type, duration of infertility and infertility diagnosis) were abstracted from electronic medical records. Infertility types included primary infertility and secondary infertility. Infertility diagnosis was classified as female factor, male factor, mix factor or unexplained reasons. Female factor infertility included diminished ovarian reserve, tubal factor, ovulatory dysfunction, endometriosis and uterine factor.

### 2.4 Statistical analysis

Descriptive information of the participants is presented as the number (%) or mean ± standard deviation where appropriate. Phthalate metabolite concentrations are presented as quartiles and geometric means. The molar sum of metabolites of DEHP (∑DEHP) was calculated by the sum of MEHP/278.34, MEOHP/292.34 and MEHHP/294.34. ∑PAEs were also calculated by the molar sum of the concentrations of the eight phthalate metabolites detected in this study. Phthalate metabolite concentrations were standardized by creatinine due to urine dilution and categorized into tertiles (numbered 1, 2, 3 from lowest to highest tertile). We used multivariate generalized linear models with Poisson distribution and log-link function to evaluate the association of metabolite concentrations with AFC by comparing the second and third tertiles to the first (reference category). Tertiles of phthalate metabolite concentrations were also used as continuous variables in models to evaluate the dose-response relationships between metabolites concentrations and AFC. Age (continuous), body mass index (BMI, continuous), year of study (2014 or 2016) and infertility diagnosis (female factor, male factor, mix factor or unexplained) were selected as covariates according to biological relevance or prior knowledge. Race and smoking status were not included as covariates due to the low frequencies of non-Han ethnicity (3.4%) and smokers (4.0%). Age was a critical independent impactor of AFC, to explore its potential modification effect, women were stratified into younger group (< 35 years) and older group (≥ 35 years) before analyses (33, 34). To test the strength of our results, we conducted sensitivity analyses by restricting our analyses to only women who sought infertility treatment due to tubal factors or male factors and had a normal BMI (18.5-24.9 kg/m²). As the results showed that age could modify the associations between phthalate metabolite concentrations and AFC, we further performed re-analyses based on age stratification. Statistical analysis was conducted by SPSS (version 22.0, IBM Co., Armonk, USA).

## 3 Results

This study comprised 525 women undergoing IVF who were recruited on two separate occasions, as shown in **Table 1**. A total of 110 subjects were enrolled in 2014, and 415 were enrolled in 2016. The average (± SD) age and BMI of the study population were 31.1 ± 5.1 years and 21.9 ± 2.7 kg/m², respectively, and most of them were of Han ethnicity (96.6%) and had never smoked (96.0%). The average (± SD) duration of infertility was 3.7 ± 2.9 years, and more than half of the subjects had primary infertility (54.5%) and were diagnosed with female factors (65.6%). The average (± SD) AFC was 13.4 ± 6.8. Most of the tested phthalate metabolites were detected in urine in most of the participants (> 92.6%), except for MOP (26.1%), as shown in **Table 2**. MBP was the phthalate with the highest level of exposure (median: 187 μg/L), and except for mono-benzyl phthalate (median: 0.16 μg/L), the remaining 5 metabolites had similar concentrations (median: 12.9-18.8 μg/L). MOP was removed from further analyses because of its low detection frequency.

**Table 1:** Description of the study population (N = 525).

Characteristics	Mean ± SD or N (%)
Age (years)	31.1 ± 5.1
BMI (kg/m²)	21.9 ± 2.7
Ethnicity	
Han	507 (96.6)
Other	18 (3.4)
Smoking status	
Ever	21 (4.0)
Never	504 (96.0)
Year of study	
2014	110 (21.0)
2016	415 (79.0)
AFC	13.4 ± 6.8
Duration of infertility (years)	3.7 ± 2.9
Infertility type	
Primary infertility	286 (54.5)
Secondary infertility	239 (45.5)
Infertility diagnosis	
Female factor	344 (65.5)
Tubal factor	206 (39.2)
Ovulatory dysfunction	45 (8.6)
Diminished ovarian reserve	56 (10.7)
Endometriosis	24 (4.6)
Uterine factor	13 (2.5)
Male factor	76 (14.5)
Mix factor	86 (16.4)
Unexplained	19 (3.6)

**Table 2:** Distributions of urinary phthalate metabolite concentrations (μg/L).

Metabolites	LOD	% > LOD	GM	25th	50th	75th
MMP	0.03	92.6	9.19	5.11	12.9	29.4
MEP	0.02	100	16.5	6.83	14.6	34.2
MBP	0.01	100	161	82.0	187	350
MBzP	0.01	96.4	0.18	0.05	0.16	0.85
MEHP	0.02	98.7	12.2	5.73	13.5	29.7
MEHHP	0.01	100	18.2	10.1	18.5	31.4
MEOHP	0.01	100	13.3	6.96	13.8	23.8
MOP	0.04	26.1	0.05	< LOD	< LOD	0.05
∑DEHP^a^	–	–	0.17	0.08	0.17	0.30
∑PAEs^a^	–	–	1.35	0.77	1.42	2.65

LOD, limit of detection; GM, geometric mean.

a ∑DEHP and ∑PAEs were expressed in μmol/L.

**Figure 1:**
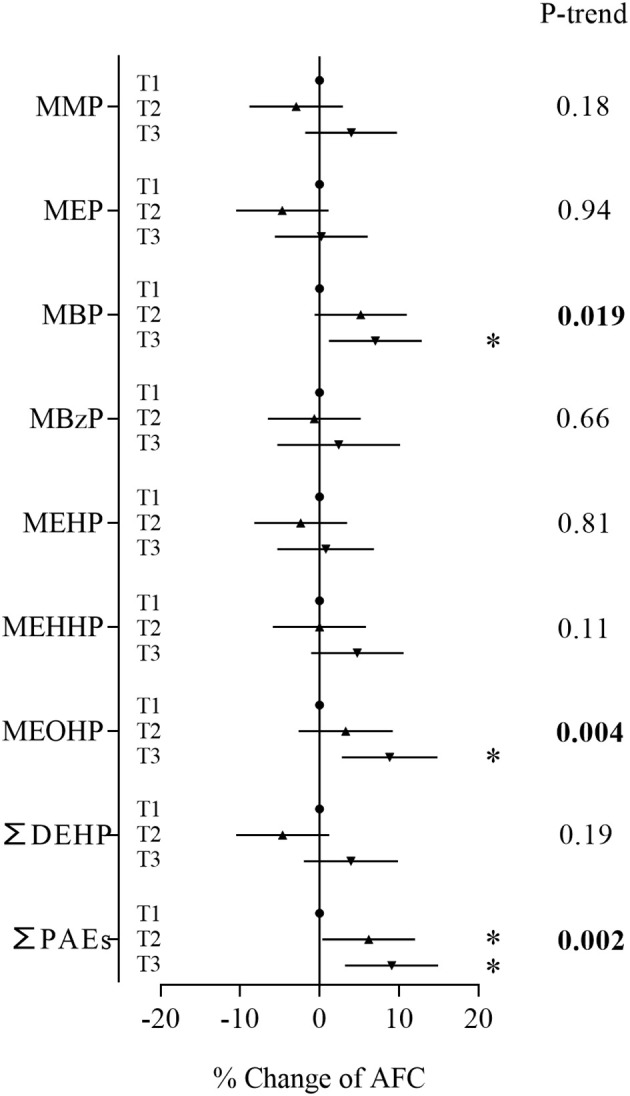
Associations between phthalate metabolite concentrations and AFC. Models were adjusted for age, BMI, year of study and infertility diagnosis. *P < 0.05.

In the Poisson regression models adjusted for age, BMI, year of study and infertility diagnosis, we found that concentrations of MBP, MEOHP and ∑PAEs were positively associated with AFC (**Figure 1**). Compared with women in the first tertile, women in the third tertile of MBP and MEOHP had a 7.02% (95% CI: 1.18%, 12.9%) and 8.84% (95% CI: 2.83%, 14.9%) increase in AFC, respectively, and women in the second and third tertiles of ∑PAEs had a 6.19% (95% CI: 0.37%, 12.0%) and 9.09% (95% CI: 3.22%, 15.0%) increase in AFC, respectively (**Supplementary Table S1**). In trend tests by tertile for the dose-response relationship, MBP, MEOHP and ∑PAEs also showed significant positive associations with AFC.

**Figure 2:**
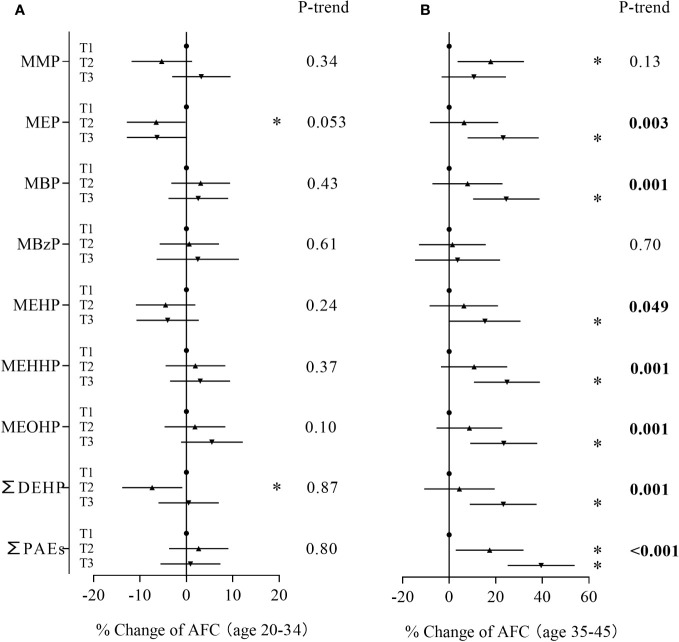
Associations between phthalate metabolite concentrations and AFC stratified by age. **(A)**: women < 35 years, **(B)**: women ≥ 35 years. *P < 0.05.

In the age-stratified analysis, we observed a stronger relationship between phthalate metabolite concentrations and AFC among women ≥ 35 years and inverse associations among women less than 35 years (**Figure 2**). Compared to women in the first tertile, younger women in the second tertile of MEP and ∑DEHP had 6.50% (95% CI: -12.8%, -0.18%) and 7.37% (95% CI: -13.8%, -0.89%) lower AFC, respectively (**Supplementary Table S2**). However, trend tests for dose-response were not significant. Among older women, subjects in the second tertile of mono-methyl phthalate and ∑PAEs and the third tertile of MEP, MBP, MEHP, MEHHP, MEOHP, ∑DEHP and ∑PAEs had a 15.3% to 39.5% increase in AFC when compared with women in the first tertile (**Supplementary Table S3**). In addition, MEP, MBP, MEHP, MEHHP, MEOHP, ∑DEHP and ∑PAEs also showed significant trends. To explore whether the discrepancies were caused by differences in phthalate exposure levels, we compared phthalate metabolite concentrations between the two groups. The results showed that younger women and older women had similar phthalate exposure levels (**Supplementary Table S4**).

**Figure 3:**
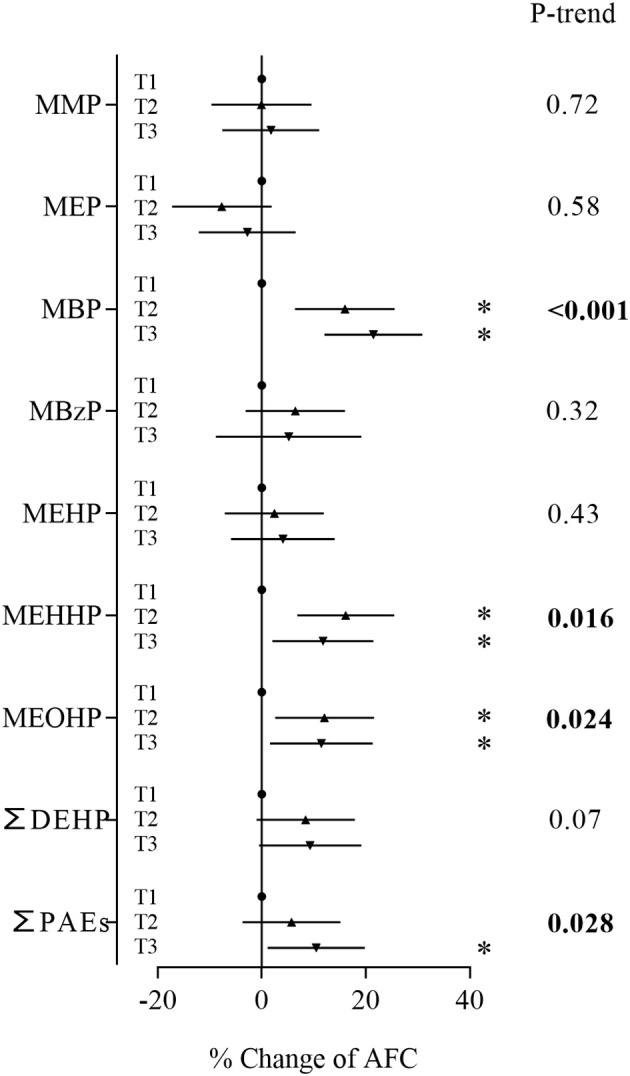
Associations between phthalate metabolite concentrations and AFC based on women diagnosed with tubal factor and infertility due to male factor. *P < 0.05.

**Figure 4:**
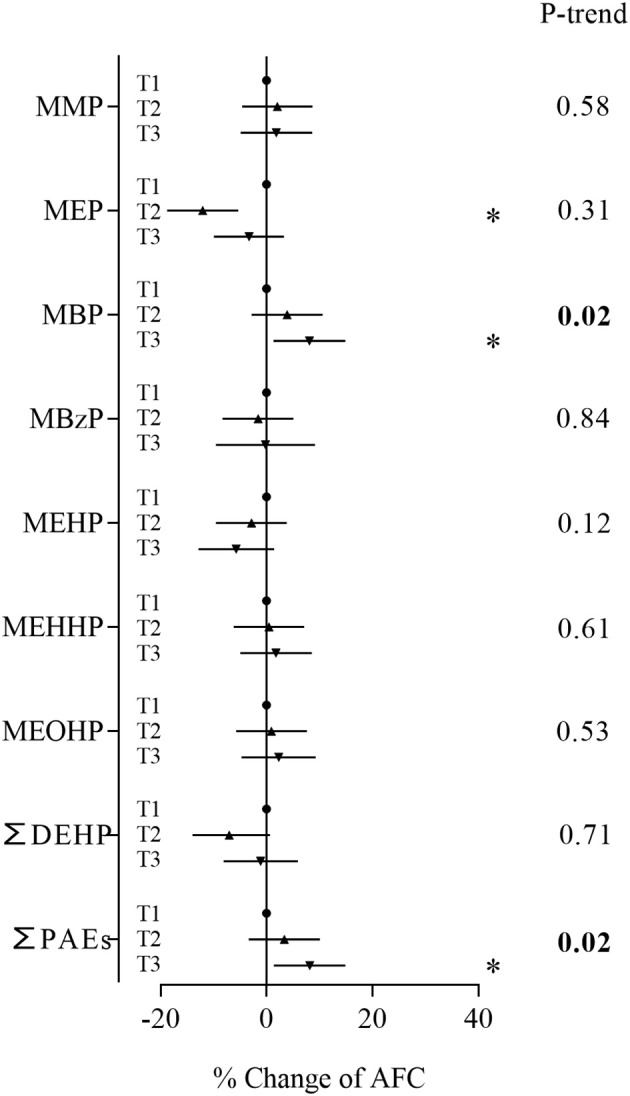
Associations between phthalate metabolite concentrations and AFC based on women with normal BMI. *P < 0.05.

In the sensitivity analyses based on women who were diagnosed with tubal factor infertility and infertility due to male factor, MBP, the major contaminant, and sum of PAEs showed positive associations with AFC (**Figure 3**). Women in the second and third tertiles of MBP and third tertile of ∑PAEs had an AFC range from 9.48% to 12.2% higher than women in the first tertile (**Supplementary Table S5**). These metabolites also showed consistent significant positive associations with AFC in trend tests. In the sensitivity analyses based on women with normal BMI, MBP and the sum of PAEs still showed positive associations with AFC (**Figure 4**; **Supplementary Table S8**). Similarly, stronger relationships were observed between phthalate metabolite concentrations and AFC among women ≥ 35 years than women < 35 years in all sensitivity analyses (**Supplementary Tables S6**, **S7**, **S9**, **S10**).

## 4 Discussion

In this study, we found that most of the phthalate metabolites were highly detected in urine samples, and MBP had the highest concentration. The European Food Safety Authorities have recommended the tolerable daily intake for several phthalates, as 10 and 50 μg/kg bw/day for DBP and DEHP, respectively (35). Estimated daily intakes for DBP and DEHP were calculated as reported (36), 58.7% and 10.3% of subjects had higher estimated daily intakes than the tolerable daily intake for DBP and DEHP, respectively, in this study. Compared with previous studies based on 125 non-pregnant women (37) and 946 pregnant women (38) in the same region, the detection rate was similar, and MBP uniformly showed the highest concentration, followed by DEHP metabolites, while the values were higher in our study (median: MBP 187 vs. 62.1 and 41.8 μg/L, MEHHP 18.5 vs. 6.34 and 5.05 μg/L, MEOHP 13.8 vs. 4.74 and 3.99 μg/L, MEHP 13.5 vs. 2.15 and 2.11 μg/L, respectively). This result may indicate an association between phthalate exposure and women with infertility.

We found that urinary MBP, MEOHP and ∑PAEs concentrations were positively associated with AFC, and most of the relationships remained in sensitivity analyses, which suggests the robustness of our results. In line with a previous study, Li et al. reported that the serum concentration of MEHHP was associated with an increased AFC among 297 IVF women (25). However, in a study of 215 women who sought infertility care, Messerlian et al. found a nonlinear inverse association between urinary DEHP metabolite concentrations (MEHP, MEOHP, MEHHP and MECPP) and AFC (24). These discrepancies may be attributed to differences in demographic characteristics (e.g., age, BMI, race, etc.). Furthermore, phthalate exposure status varied between studies. In Messerlian’s study, MEP showed the highest concentration, while the concentration of MBP was relatively low (SG-adjusted median: 54.2 and 12.8 μg/L, respectively), which was in contrast with our data (median: 14.6 and 187 μg/L, respectively). The overall metabolite concentrations were low in Li’s study (median: MEP 2.19 μg/L, MBP 4.17 μg/L). In addition, phthalate may exert toxicity with a nonmonotonic dose-response effect or even a U-shape effect (39).

Primordial follicles, which are composed of a prophase-arrested oocyte enclosed by a single layer of flattened pre-granulosa cells, form at approximately 15-22 weeks of gestation and are complete by 6 months after birth in the human ovary (40). Their fates were to remain dormant, awaken and develop, or die directly during dormancy (41). Only a few of the dormant primordial follicles are activated under the regulation of intercellular and intracellular signals. Once awakened, primordial follicles join the follicle growing pool; most of them undergo atresia during development, and a small proportion undergo consecutive development through primary follicles, secondary follicles, antral follicles, preovulatory follicles and ovulation (42, 43). Therefore, a greater AFC indicates that more primordial follicles were awakened originally. In support of our findings, it has been reported that DEHP accelerates primordial follicle recruitment by decreasing the percentage of primordial follicles and increasing the percentage of developing follicles in postnatal mice (27) and in adult mice (28). Exposure to di-n-butyl phthalate, the prototype of MBP, promotes the depletion of follicular follicles by accelerating primordial follicle recruitment in rats (29). Additionally, in an *in vitro* culture neonatal ovary model, Hannon et al. revealed that MEHP could directly accelerate primordial follicle recruitment by over activating PI3K signals (44).

The number of primordial follicles in the ovary determines the ovarian reserve (40). Any factors that accelerate primordial follicle recruitment will promote the depletion of ovarian reserve and shorten the reproductive life span of women. It has been reported that phthalate exposure was associated with the risk of premature ovarian failure (45). On the other hand, although a higher AFC was observed in women with higher urinary phthalate metabolite concentrations in this study, follicle development competence may be affected. Previous studies found that phthalate could increase oocyte oxidative stress, disrupt the cell cycle, impair meiotic competence, induce oocyte and granulosa cell apoptosis, inhibit oocyte maturation, cause epigenetic alterations, disrupt DNA damage repair gene expression, etc., which may contribute to adverse reproductive outcomes (10, 46–50).

Age was a critical independent risk factor for female fecundity and was associated with adverse IVF outcomes (e.g., lower oocyte retrieval, pregnancy rate, delivery rate, etc.) (34, 51). In age stratification analyses based on all subjects and in sensitivity analyses, we found that the associations between phthalate metabolite concentrations and AFC were modified by age, which is in line with a previous study (24). In women with advanced age, the ovary may be more vulnerable to external hazardous factors such as phthalates. The risk resistance ability and repair capacity decline with ovarian ageing, such as mitochondrial DNA instability and dysfunction, disturbance of antioxidant signaling and increase in oxidative damage, DNA damage, aneuploidy, epigenetic alteration, microenvironmental alteration (52–54). Moreover, exposure duration is also an important factor. It has been widely reported that women are exposed to phthalate from the fetal period to childhood and then adulthood (38, 55, 56), and long-term exposure is more likely to lead to adverse consequences.

In summary, our results showed that phthalate metabolite concentrations in urine were positively associated with AFC among women undergoing IVF. However, this study still has some limitations. (1) The AFC was measured when women attended the infertility clinic for ovarian reserve assessment, while urine samples were collected the on day of surgery, which was up to 4 months after AFC measurement, and the median interval between AFC measurement and sample collection was 72 days. Humans are continuously exposed to phthalates, and it has been reported that a spot urine sample was a moderate predictor of the 4-month exposure level of phthalates (57). (2) The study population was enrolled from a reproductive center, and they had higher phthalate exposure levels than the normal population as described above; therefore, these findings may not be applicable to the general population. (3) It is important to note that phthalate exposure is just one of the factors that can influence reproductive health and fertility. Lifestyle, genetics, and other environmental factors also play a role, we cannot rule out the influences from other factors in this study. (4) We found positive associations between phthalate exposure and AFC, and while the causation cannot be determined, more studies are needed to better understand their relationships.

## 5 Conclusions

The results support the idea that phthalate exposure could accelerate primordial follicle recruitment and promote the depletion of ovarian reserve. Our research fills the gap between animal studies showing that phthalates accelerate primordial follicle recruitment and epidemiological research showing that phthalates are associated with premature ovarian failure. Considering the high exposure frequency and level of phthalates in infertile women, more studies are needed to determine the effect of phthalate on ovarian reserve and folliculogenesis.

## Data availability statement

The original contributions presented in the study are included in the article/**Supplementary Material**, further inquiries can be directed to the corresponding author/s.

## Ethics statement

The studies involving humans were approved by Ethics Board of Tongji Hospital. The studies were conducted in accordance with the local legislation and institutional requirements. The participants provided their written informed consent to participate in this study.

## Author contributions

YY: Formal Analysis, Investigation, Methodology, Writing – original draft. YD: Data curation, Investigation, Resources, Writing – review & editing. NG: Investigation, Resources, Writing – review & editing. FL: Supervision, Writing – review & editing. TD: Investigation, Methodology, Writing – review & editing. YL: Funding acquisition, Resources, Supervision, Writing – review & editing.
