# Supplementary material for: Associations between urinary phthalate concentrations and antral follicle count among women undergoing in vitro fertilization
Source: Front Endocrinol (Lausanne). 2024 Jan 8;14:1286391. doi: 10.3389/fendo.2023.1286391 (PMC10801055; doi:10.3389/fendo.2023.1286391)
Supplement: Supplementary file 1 [file 1286391_Table_1.docx]

**Supplementary Materials**

**Associations between urinary phthalate concentrations and antral follicle count among women undergoing in vitro fertilization**

Yangcheng Yao, Yaoyao Du, Na Guo, Fenghua Liu, Taoran Deng, Yufeng Li

**Table of Contents**

**Table S1**. Associations between phthalate metabolite concentrations and AFC.

**Table S2**. Associations between phthalate metabolite concentrations and AFC in younger women.

**Table S3**. Associations between phthalate metabolite concentrations and AFC in older women.

**Table S4**. Comparison of phthalate metabolite concentrations between younger and older women.

**Table S5**. Associations between phthalate metabolite concentrations and AFC based on women diagnosed with tubal factor and infertility due to male factor.

**Table S6**. Associations between phthalate metabolite concentrations and AFC based on younger women diagnosed with tubal factor and infertility due to male factor.

**Table S7**. Associations between phthalate metabolite concentrations and AFC based on older women diagnosed with tubal factor and infertility due to male factor.

**Table S8**. Associations between phthalate metabolite concentrations and AFC based on women with normal BMI.

**Table S9**. Associations between phthalate metabolite concentrations and AFC based on younger women with normal BMI.

**Table S10**. Associations between phthalate metabolite concentrations and AFC based on older women with normal BMI.

| **Table S1**. Associations between phthalate metabolite concentrations and AFC (N = 525). | | | | |
| --- | --- | --- | --- | --- |
| Metabolites | Percent change (95% CI) of AFC | | | P-trend |
|  | T1 | T2 | T3 |  |
| MMP | Ref | -2.95 (-8.83, 2.93) | 3.98 (-1.79, 9.75) | 0.18 |
| MEP | Ref | -4.70 (-10.5, 1.12) | 0.23 (-5.60, 6.05) | 0.94 |
| MBP | Ref | 5.18 (-0.63, 11.0) | **7.02 (1.18, 12.9)** | **0.02** |
| MBzP | Ref | -0.65 (-6.48, 5.18) | 2.41 (-5.33, 10.2) | 0.66 |
| MEHP | Ref | -2.38 (-8.21, 3.45) | 0.77 (-5.30, 6.85) | 0.81 |
| MEHHP | Ref | -0.02 (-5.87, 5.82) | 4.76 (-1.06, 10.6) | 0.11 |
| MEOHP | Ref | 3.28 (-2.63, 9.20) | **8.84 (2.83, 14.9)** | **0.004** |
| ∑DEHP | Ref | -4.64 (-10.5, 1.23) | 3.95 (-1.96, 9.87) | 0.19 |
| ∑PAEs | Ref | **6.19 (0.37, 12.0)** | **9.09 (3.22, 15.0)** | **0.002** |
| Bold indicates that the compared items have statistical significance (P < 0.05). | | | | |
| Models were adjusted for age, BMI, year of study and infertility diagnosis. | | | | |

| **Table S2**. Associations between phthalate metabolite concentrations and AFC in younger women (Age < 35 years, N = 392). | | | | |
| --- | --- | --- | --- | --- |
| Metabolites | Percent change (95% CI) of AFC | | | P-trend |
|  | T1 | T2 | T3 |  |
| MMP | Ref | -5.30 (-11.8, 1.21) | 3.22 (-3.09, 9.54) | 0.34 |
| MEP | Ref | **-6.50 (-12.8, -0.18)** | -6.32 (-12.8, 0.15) | 0.053 |
| MBP | Ref | 3.09 (-3.27, 9.45) | 2.56 (-3.87, 8.99) | 0.43 |
| MBzP | Ref | 0.62 (-5.76, 7.01) | 2.45 (-6.41, 11.3) | 0.61 |
| MEHP | Ref | -4.46 (-10.9, 1.95) | -4.03 (-10.7, 2.68) | 0.24 |
| MEHHP | Ref | 1.93 (-4.50, 8.37) | 2.98 (-3.50, 9.46) | 0.37 |
| MEOHP | Ref | 1.85 (-4.70, 8.39) | 5.50 (-1.14, 12.2) | 0.10 |
| ∑DEHP | Ref | **-7.37 (-13.8, -0.89)** | 0.49 (-6.02, 6.99) | 0.87 |
| ∑PAEs | Ref | 2.65 (-3.74, 9.05) | 0.88 (-5.57, 7.32) | 0.80 |
| Bold indicates that the compared items have statistical significance (P < 0.05). | | | | |
| Models were adjusted for age, BMI, year of study and infertility diagnosis. | | | | |

| **Table S3**. Associations between phthalate metabolite concentrations and AFC in older women (Age ≥ 35 years, N = 133). | | | | |
| --- | --- | --- | --- | --- |
| Metabolites | Percent change (95% CI) of AFC | | | P-trend |
|  | T1 | T2 | T3 |  |
| MMP | Ref | **17.8 (3.56, 32.1)** | 10.6 (-3.27, 24.4) | 0.13 |
| MEP | Ref | 6.39 (-8.30, 21.1) | **23.1 (7.89, 38.4)** | **0.003** |
| MBP | Ref | 7.84 (-7.19, 22.9) | **24.5 (10.3, 38.8)** | **0.001** |
| MBzP | Ref | 1.34 (-13.0, 15.7) | 3.60 (-14.7, 21.9) | 0.70 |
| MEHP | Ref | 6.27 (-8.46, 21.0) | **15.3 (0.03, 30.7)** | **0.049** |
| MEHHP | Ref | 10.7 (-3.53, 25.0) | **24.8 (10.6, 39.0)** | **0.001** |
| MEOHP | Ref | 8.71 (-5.43, 22.9) | **23.4 (9.06, 37.8)** | **0.001** |
| ∑DEHP | Ref | 4.40 (-10.8, 19. 6) | **23.2 (8.86, 37.6)** | **0.001** |
| ∑PAEs | Ref | **17.4 (2.80, 32.0)** | **39.5 (25.1, 53.9)** | **<0.001** |
| Bold indicates that the compared items have statistical significance (P < 0.05). | | | | |
| Models were adjusted for age, BMI, year of study and infertility diagnosis. | | | | |

| **Table S4**. Comparison of phthalate metabolite concentrations between younger and older women^a^ (μg/g Cr). | | | | | | |
| --- | --- | --- | --- | --- | --- | --- |
| Metabolites | Age < 35 (N = 392) | |  | Age ≥ 35 (N = 133) | | P^b^ |
|  | GM | Median |  | GM | Median |  |
| MMP | 6.03 | 7.50 |  | 7.64 | 6.76 | 0.88 |
| MEP | 11.8 | 9.17 |  | 10.8 | 9.69 | 0.44 |
| MBP | 113 | 120 |  | 110 | 120 | 0.90 |
| MBzP | 0.12 | 0.10 |  | 0.13 | 0.11 | 0.76 |
| MEHP | 8.14 | 9.38 |  | 9.76 | 9.68 | 0.19 |
| MEHHP | 12.3 | 11.3 |  | 13.8 | 12.8 | 0.16 |
| MEOHP | 9.15 | 9.32 |  | 9.75 | 8.49 | 0.93 |
| ∑DEHP^b^ | 0.11 | 0.11 |  | 0.13 | 0.11 | 0.26 |
| ∑PAEs^b^ | 0.92 | 0.91 |  | 1.02 | 1.06 | 0.16 |
| Abbreviations: GM, geometric mean; Cr, creatinine. | | | | | | |
| ^a^: Compared by Mann-Whitney U test. | | | | | | |
| ^b^: ∑DEHP and ∑PAEs were expressed in μmol/g Cr. | | | | | | |

| **Table S5**. Associations between phthalate metabolite concentrations and AFC based on women diagnosed with tubal factor and infertility due to male factor (N = 282). | | | | |
| --- | --- | --- | --- | --- |
| Metabolites | Percent change (95% CI) of AFC | | | P-trend |
|  | T1 | T2 | T3 |  |
| MMP | Ref | -2.50 (-10.5, 5.53) | 2.35 (-5.42, 10.1) | 0.56 |
| MEP | Ref | 1.34 (-6.50, 9.17) | 1.54 (-6.36, 9.44) | 0.70 |
| MBP | Ref | **9.48 (1.57, 17.4)** | **12.2 (4.20, 20.1)** | **0.003** |
| MBzP | Ref | 4.96 (-2.96, 12.9) | 3.08 (-8.85, 15.0) | 0.40 |
| MEHP | Ref | -0.75 (-8.64, 7.14) | -1.02 (-9.46, 7.43) | 0.81 |
| MEHHP | Ref | 5.80 (-1.98, 13.6) | 4.44 (-3.57, 12.5) | 0.27 |
| MEOHP | Ref | 4.83 (-3.07, 12.7) | 4.29 (-3.95, 12.5) | 0.31 |
| ∑DEHP | Ref | 0.91 (-6.97, 8.78) | 3.57 (-4.61, 11.7) | 0.39 |
| ∑PAEs | Ref | 5.37 (-2.48, 13.2) | **10.3 (2.49, 18.1)** | **0.01** |
| Bold indicates that the compared items have statistical significance (P < 0.05). | | | | |
| Models were adjusted for age, BMI, year of study and infertility diagnosis. | | | | |

| **Table S6**. Associations between phthalate metabolite concentrations and AFC based on younger women diagnosed with tubal factor and infertility due to male factor (Age < 35 years, N = 221). | | | | |
| --- | --- | --- | --- | --- |
| Metabolites | Percent change (95% CI) of AFC | | | P-trend |
|  | T1 | T2 | T3 |  |
| MMP | Ref | -3.01 (-11.6, 5.63) | -1.99 (-10.4, 6.40) | 0.63 |
| MEP | Ref | -0.42 (-8.90, 8.06) | -7.97 (-16.6, 0.65) | 0.07 |
| MBP | Ref | **11.5 (2.92, 20.0)** | **14.0 (5.54, 22.5)** | **0.001** |
| MBzP | Ref | 3.22 (-5.35, 11.8) | 1.79 (-10.8, 14.4) | 0.64 |
| MEHP | Ref | -2.57 (-11.1, 5.97) | -0.94 (-9.88, 7.99) | 0.84 |
| MEHHP | Ref | 5.11 (-3.30, 13.5) | 4.35 (-4.27, 13.0) | 0.32 |
| MEOHP | Ref | 2.12 (-6.48, 10.7) | 5.56 (-3.30, 14.4) | 0.22 |
| ∑DEHP | Ref | -2.61 (-11.2, 5.96) | 3.54 (-5.19, 12.3) | 0.42 |
| ∑PAEs | Ref | 5.02 (-3.43, 13.5) | 6.23 (-2.18, 14.6) | 0.15 |
| Bold indicates that the compared items have statistical significance (P < 0.05). | | | | |
| Models were adjusted for age, BMI, year of study and infertility diagnosis. | | | | |

| **Table S7**. Associations between phthalate metabolite concentrations and AFC based on older women diagnosed with tubal factor and infertility due to male factor (Age ≥ 35 years, N = 61). | | | | |
| --- | --- | --- | --- | --- |
| Metabolites | Percent change (95% CI) of AFC | | | P-trend |
|  | T1 | T2 | T3 |  |
| MMP | Ref | 18.7 (-5.39, 42.7) | 19.8 (-3.17, 42.8) | 0.10 |
| MEP | Ref | 9.26 (-15.0, 33.5) | **35.3 (12.5, 58.0)** | **0.002** |
| MBP | Ref | 11.1 (-15.7, 37.8) | **31.1 (3.31, 58.9)** | **0.02** |
| MBzP | Ref | **25.0 (0.95, 49.1)** | 1.45 (-34.3, 37.2) | 0.38 |
| MEHP | Ref | **28.5 (4.78, 52.2)** | **31.7 (4.49, 58.8)** | **0.02** |
| MEHHP | Ref | **40.0 (17.3, 62.7)** | 17.8 (-8.37, 43.9) | 0.10 |
| MEOHP | Ref | **29.8 (7.75, 51.8)** | 2.85 (-23.5, 29.2) | 0.57 |
| ∑DEHP | Ref | **27.9 (3.87, 52.0)** | 12.2 (-14.0, 38.4) | 0.33 |
| ∑PAEs | Ref | 17.8 (-7.16, 42.7) | **39.2 (15.2, 63.2)** | **0.001** |
| Bold indicates that the compared items have statistical significance (P < 0.05). | | | | |
| Models were adjusted for age, BMI, year of study and infertility diagnosis. | | | | |

| **Table S8**. Associations between phthalate metabolite concentrations and AFC based on women with normal BMI (N = 404). | | | | |
| --- | --- | --- | --- | --- |
| Metabolites | Percent change (95% CI) of AFC | | | P-trend |
|  | T1 | T2 | T3 |  |
| MMP | Ref | 2.01 (-4.63, 8.65) | 1.85 (-4.90, 8.59) | 0.58 |
| MEP | Ref | **-12.1 (-18.8, -5.35)** | -3.32 (-9.94, 3.29) | 0.31 |
| MBP | Ref | 3.88 (-2.85, 10.6) | **8.07 (1.29, 14.9)** | **0.02** |
| MBzP | Ref | -1.61 (-8.28, 5.05) | -0.21 (-9.53, 9.12) | 0.84 |
| MEHP | Ref | -2.87 (-9.57, 3.83) | -5.71 (-12.8, 1.41) | 0.12 |
| MEHHP | Ref | 0.47 (-6.17, 7.11) | 1.78 (-4.98, 8.54) | 0.61 |
| MEOHP | Ref | 0.94 (-5.76, 7.65) | 2.27 (-4.75, 9.29) | 0.53 |
| ∑DEHP | Ref | -7.33 (-14.0, 0.66) | -1.10 (-8.10, 5.89) | 0.71 |
| ∑PAEs | Ref | 3.36 (-3.34, 10.1) | **8.13 (1.38, 14.9)** | **0.02** |
| Bold indicates that the compared items have statistical significance (P < 0.05). | | | | |
| Models were adjusted for age, BMI, year of study and infertility diagnosis. | | | | |

| **Table S9**. Associations between phthalate metabolite concentrations and AFC based on younger women with normal BMI (Age < 35 years, N = 299). | | | | |
| --- | --- | --- | --- | --- |
| Metabolites | Percent change (95% CI) of AFC | | | P-trend |
|  | T1 | T2 | T3 |  |
| MMP | Ref | 0.87 (-6.19, 7.93) | 0.15 (-6.97, 7.28) | 0.96 |
| MEP | Ref | **-11.1 (-18.3, -3.94)** | -5.21 (-12.2, 1.80) | 0.15 |
| MBP | Ref | 1.84 (-5.24, 8.92) | 3.76 (-3.39, 10.9) | 0.30 |
| MBzP | Ref | -6.43 (-13.5, 0.63) | -3.31 (-13.7, 7.12) | 0.23 |
| MEHP | Ref | -5.95 (-13.1, 1.16) | **-11.0 (-18.6, -3.36)** | **0.01** |
| MEHHP | Ref | 0.75 (-6.30, 7.80) | -0.08 (-7.25, 7.08) | 0.98 |
| MEOHP | Ref | -2.11 (-9.25, 5.03) | -0.18 (-7.64, 7.28) | 0.96 |
| ∑DEHP | Ref | **-8.36 (-15.5, -1.25)** | -5.40 (-12.8, 1.97) | 0.14 |
| ∑PAEs | Ref | -0.07 (-7.19, 7.04) | 3.45 (-3.71, 10.6) | 0.34 |
| Bold indicates that the compared items have statistical significance (P < 0.05). | | | | |
| Models were adjusted for age, BMI, year of study and infertility diagnosis. | | | | |

| **Table S10**. Associations between phthalate metabolite concentrations and AFC based on older women with normal BMI (Age ≥ 35 years, N = 105). | | | | |
| --- | --- | --- | --- | --- |
| Metabolites | Percent change (95% CI) of AFC | | | P-trend |
|  | T1 | T2 | T3 |  |
| MMP | Ref | **17.5 (1.42, 33.7)** | 12.0 (-4.56, 28.6) | 0.14 |
| MEP | Ref | 5.37 (-11.6, 22.3) | 11.2 (-6.44, 28.9) | 0.21 |
| MBP | Ref | 15.3 (-2.62, 33.3) | **28.6 (11.7, 45.6)** | **0.001** |
| MBzP | Ref | -3.43 (-20.1, 13.2) | 5.47 (-14.3, 25.2) | 0.70 |
| MEHP | Ref | 12.5 (-5.26, 30.2) | 18.1 (-0.10, 36.4) | 0.05 |
| MEHHP | Ref | 16.9 (-0.12, 33.9) | **24.3 (7.96, 40.7)** | **0.004** |
| MEOHP | Ref | 16.3 (-0.45, 33.1) | **26.7 (10.3, 43.2)** | **0.001** |
| ∑DEHP | Ref | **20.6 (2.80, 38.4)** | **29.7 (12.8, 46.6)** | **0.001** |
| ∑PAEs | Ref | **26.8 (9.31, 44.3)** | **45.4 (28.4, 62.5)** | **<0.001** |
| Bold indicates that the compared items have statistical significance (P < 0.05). | | | | |
| Models were adjusted for age, BMI, year of study and infertility diagnosis. | | | | |
